# ChemSkin Reference Chemical Database for the Development of an In Vitro Skin Irritation Test

**DOI:** 10.3390/toxics9110314

**Published:** 2021-11-18

**Authors:** Juhee Han, Ga-Young Lee, Green Bae, Mi-Jeong Kang, Kyung-Min Lim

**Affiliations:** 1 College of Pharmacy, Ewha Womans University, Seoul 03760, Korea; hanju1996@naver.com (J.H.); ryoungi22@korea.kr (G.-Y.L.); greeni77@gmail.com (G.B.); 2 Korea Water Resources Environment Institute, Gyeongsan 38650, Korea

**Keywords:** skin irritation test, reference chemical, alternative test method, chemical database

## Abstract

Since the animal test ban on cosmetics in the EU in 2013, alternative in vitro safety tests have been actively researched to replace in vivo animal tests. For the development and evaluation of a new test method, reference chemicals with quality in vivo data are essential to assess the predictive capacity and applicability domain. Here, we compiled a reference chemical database (ChemSkin DB) for the development and evaluation of new in vitro skin irritation tests. The first candidates were selected from 317 chemicals (source data *n* = 1567) searched from the literature from the last 20 years, including previous validation study reports, ECETOC, and published papers. Chemicals showing inconsistent classification or those that were commercially unavailable, difficult or dangerous to handle, prohibitively expensive, or without quality in vivo or in vitro data were removed, leaving a total of 100 chemicals. Supporting references, in vivo Draize scores, UN GHS/EU CLP classifications and commercial sources were compiled. Test results produced by the approved methods of OECD Test No. 439 were included and compared using the classification table, scatter plot, and Pearson correlation analysis to identify the false predictions and differences between in vitro skin irritation tests. These results may provide an insight into the future development of new in vitro skin irritation tests.

## 1. Introduction

Cosmetics and toiletries are the main sources of human exposure to potentially dangerous chemicals among the general public [1]. Therefore, it is essential to evaluate the toxicity of chemicals used in these products before product release into market. Most of the toxicity items required for the safety evaluation of chemicals use experimental animals, but more than 35 countries have banned animal tests for cosmetics since 2013 [2,3]. Various alternatives have been developed to replace animal testing for cosmetics, including tests in nonvertebrate animals and in vitro, and in chemico and in silico methods [4,5,6]. For the regulatory body to accept the data produced from methods other than the standard test method, the methods should be verified in their ability to address the toxic endpoint with relevance and reliability to the same extent as the existing standard test method through officially endorsed procedures. This validation procedure is clearly described in OECD Guidance Document 34 [7].

Relevance, also called predictive capacity, is evaluated by comparing the concordance of the prediction for reference chemicals made by the new test method with that from the existing standard test method, generally an in vivo animal test [8,9]. Reference chemicals with quality in vivo data are, therefore, important for evaluating the relevance of new test methods [10]. A reference chemical database for in vivo eye irritation test sensitization has been well established [11]. However, to the best of our knowledge, a reference chemical database for the skin irritation test with in vivo and in vitro evidence of sufficient quality is lacking.

**Figure 1:**
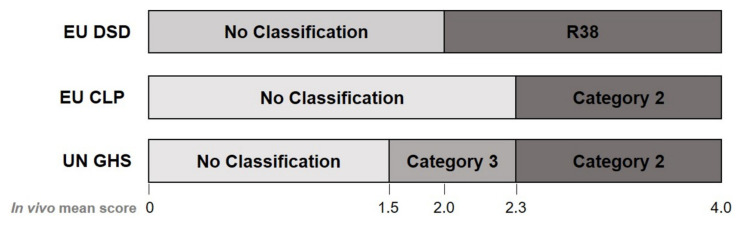
Erythema/edema in vivo Draize scores and classification of irritancy of EU DSD, EU CLP, and UN GHS. Erythema/edema Draize score ranges defining EU DSD, EU CLP, and UN GHS classification for skin irritation hazard.

The in vivo skin irritation test has been the primary test to replace, since rabbits, the test subject animal, experience an enormous level of pain and discomfort from the confinement and hair-shaving [12]. The Draize skin irritation test (OECD TG 404) [13] was developed by John Draize in 1944 [14]. In this test, the dorsal hair is shaved, and on the following day a test chemical (0.5 g solid or 0.5 mL liquid) is applied on a small area (~6 cm^2^); the treated site is covered with a patch to prevent the animal from licking off the test chemical. The patch is removed after 4 h and skin reactions are scored for signs of erythema and edema at 1, 24, 48, and 72 h. Erythema and edema are scored with grades from 0 to 4 depending on the severity. Based on the severity and reversibility, skin corrosion and irritation are categorized into Category 1, with sub-categories of 1A, 1B, and 1C (corrosive, mean scores above 4.0); Category 2 (irritant, mean scores of 2.3~4.0 for erythema or for edema in at least 2 of 3 tested animals); Category 3 (mild irritant, mean scores of 1.5~2.3 for erythema or for edema in at least 2 of 3 tested animals); or No category (below 1.5) according to the UN GHS classification. There have been multiple classification standards for skin irritation, such as the four levels of irritancy classification on primary irritation index (PII), EU Dangerous Substances Directive/Dangerous Preparations Directive (EU DSD/DPD), and EU Classification, Labelling, and Packaging Regulation (EU CLP) systems. Importantly, these classification systems are not compatible with UN GHS classification (Figure 1), which leads to uncertainty on the irritancy decision of some borderline chemicals. Therefore, it is necessary to provide both in vivo Draize scores and corresponding UN GHS classifications for reference chemicals [15,16,17].

The OECD Test No. 439: In vitro Skin Irritation: Reconstructed Human Epidermis (RhE) Test Method [18] was developed to identify No category chemicals from other chemicals in accordance with the UN GHS classification. The OECD test guideline (TG) 439 provides an in vitro procedure using RhE to predict the skin hazard of irritant chemicals (substances and mixtures) based solely on the cell viability value obtained with 3-(4,5-dimethylthiazol-2-yl)-2,5-diphenyltetrazolium bromide (MTT) assay. Three validated reference methods (VRMs), the EpiDerm™ skin irritation test (SIT), EpiSkin™ SIT, and SkinEthic RhE SIT, were originally approved for OECD TG 439 in 2010. Following the performance standards of TG 439, four me-too models were additionally approved for OECD TG 439 in 2021 [19] and new models like USP-RhE [20] are under development for inclusion in TG 439. However, TG 439 has several limitations. TG 439 does not classify chemicals to the optional UN GHS Category 3 (mild irritants). In addition, TG 439 RhE test methods cannot resolve between UN GHS Categories 1 and 2; thus, further information on skin corrosion is required to decide on the final classification of certain chemicals. To resolve these limitations, inclusion of an IL-1α assay was considered [21,22], but it failed to exceed the result of MTT assay [23]. Therefore, a novel skin irritation test method is still in demand to overcome the limitations of the current OECD TG 439.

One of the major problems for TG 439 is that incongruent sets of reference chemicals have been used to evaluate the predictive capacity of the individual RhE methods in TG 439, suggesting a need to establish a well-characterized reference chemical database for the evaluation of novel skin irritation test methods [24] that will overcome the current TG 439. Here, we compiled a reference chemical database (ChemSkin DB) for the development and evaluation of alternative methods for in vivo skin irritation test. The first candidates were selected from 317 chemicals (source data *n* = 1567) searched from the literature for the last 20 years, including previous validation study reports, ECETOC, and published papers. Chemicals without GHS category information or only with the information on past EU classification criteria were removed. In addition, chemicals showing inconsistent classification results and those without both in vivo and in vitro test results were excluded, leaving a total of 100 chemicals. Supporting references, in vivo Draize scores, UN GHS/EU CLP classifications, and commercial sources in the manuscripts were compiled. Furthermore, test results produced by OECD TG 439 (RhE Test Method) were included if available, to provide an insight into the future development of alternatives to the in vivo skin irritation test.

## 2. Materials and Methods

### 2.1. Classification Criteria for the Skin Irritancy of Chemicals

#### 2.1.1. Classification Based on Primary Irritation Index (PII)

As described in OECD TG 404 [13], the in vivo skin irritation test (Draize test) is conducted and erythema and edema scores at 24- and 72-h post-exposure (or 24, 48, and 72 h, if available) are combined and averaged into a PII. According to PII, the skin irritancy of a test chemical is classified as described in Table 1 [25].

#### 2.1.2. Classification Based on EU DSD/DPD and EU CLP

According to the EU DSD/DPD criteria, the skin irritancy of a test chemical is classified as follows: an in vivo score of 2.0 or higher is classified as R38 (irritant) and a score less than 2.0 is classified as No label (non-irritant).

As a replacement for the EU DSD/DPD regulations, the CLP regulation was created as a new system that reflects the UN GHS classification and labeling regulation. This is referred to as the EU CLP or UN GHS/CLP, which is different from the current UN GHS. Although the CLP regulation was defined in 2008 as Regulation (EC) No. 1272/2008 [16], it has been used interchangeably with the classification criteria of the previous EU DSD/DPD [15]. Chemical classification and labeling systems have been completely replaced. According to this UN GHS/CLP classification, the skin irritancy of a test chemical is classified as follows: scores < 2.3 indicate No category (non-irritant) and scores > 2.3 indicate Category 2 (irritant).

#### 2.1.3. Classification Based on the Current UN GHS

In the revised version of the UN GHS 2019 [17], the skin irritancy of a test chemical is classified as follows: No category (non-irritant) in cases of <1.5, Category 3 (mild irritant) in cases of ≥1.5 and <2.3, and Category 2 (irritant) in cases of ≥2.3 and ≤4.0. Category 3 is newly adopted but only a few authorities employ it. OECD TG 439 defines Category 3 as a non-classified chemical [19].

### 2.2. Classification of Edema/Erythema of 100 Reference Chemicals Based on the In Vivo Draize Test

The in vivo scores of 100 reference chemicals of the ChemSkin DB were obtained through the literature search. ChemSkin DB chemicals were newly classified according to the in vivo classes and in vivo categories described above.

### 2.3. Comparison of In Vivo and In Vitro Data of ChemSkin DB Reference Chemicals

In vivo scores and in vitro viability values obtained from OECD TG 439 were compared for ChemSkin DB reference chemicals. The skin irritancy was identified based on in vivo scores of 2.3 or higher or with a viability cut-off of 50%. The in vivo score was compared with the viability data of VRMs and the KeraSkin™ model. The VRMs used in the analysis are EpiSkin™, SkinEthic™, RhE, EpiDerm™, and LabCyte-EPI. The results of the test methods were plotted as a scatter plot to show the data distribution, and the cut-off values were displayed to easily demonstrate the incorrect values.

The predictive capacity of VRMs and the KeraSkin™ was calculated by indexes of sensitivity, specificity, and accuracy. According to the OECD TG 439 performance standard, a sensitivity of ≥ 80%, specificity of ≥ 70%, and accuracy of ≥ 75% must be satisfied.

The Pearson correlation coefficient was obtained to confirm the correlation between in vivo score and viability. The Pearson correlation coefficient is a measure of the degree of correlation between two variables, and it is one of the most widely used measures of relationships [26,27]. It has a range of values from −1 to +1; the closer the coefficient is to the absolute value of 1, the greater the association between the two variables. Correlation coefficients ≤0.35 are generally considered to represent low or weak correlations, those of 0.36 to 0.67 have a modest or moderate correlation, those of 0.68 to 1.0 indicate a strong or high correlation, and correlation coefficients ≥ 0.90 reflect a very high correlation [28].

## 3. Results and Discussion

### 3.1. Chemical Selection for the Establishment of ChemSkin DB

**Figure 2:**
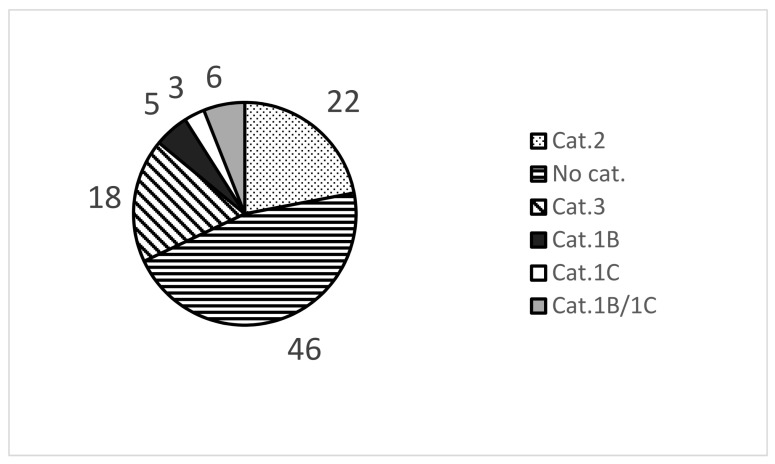
Composition of 100 reference chemicals in ChemSkin DB Pie chart of 100 chemicals classified by in the in vivo category.

**Figure 3:**
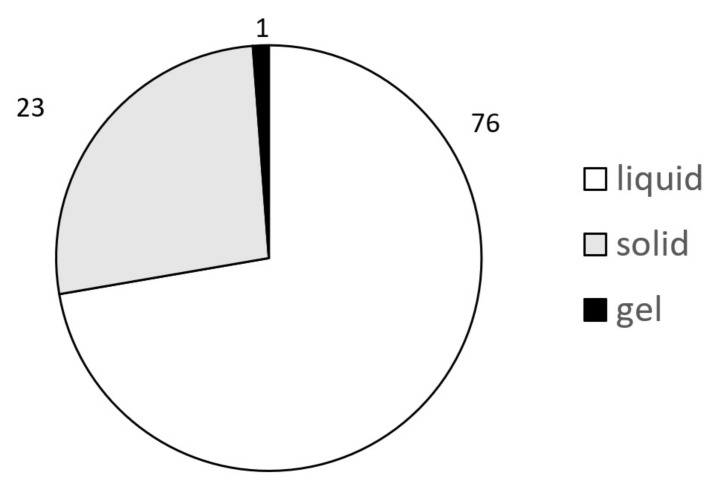
Composition of 100 reference chemicals in ChemSkin DB Pie chart of 100 chemicals classified by physical state.

To establish the ChemSkin DB, the reference chemicals were searched from the literature over the last 20 years and reviewed for the quality of in vivo data and availability of in vivo scores, which is critical for the classification of optional Category 3 as recently stated by UN GHS. In addition to in vivo results, human patch test results and the in vitro results produced by four validated reference methods of OECD 439 were included. The final ChemSkin DB was completed by adding the source literature information or official review reports (SCCS, SCCP, CIR, etc.). Through this procedure, 100 reference chemicals were included (source data *n* = 1567) in the final version of ChemSkin DB. The composition of 100 chemicals is shown in Table 2 and Figure 2 and Figure 3.

The reference chemical group included 46 No category chemicals, 18 optional Category 3 chemical, 22 Category 2 chemicals, 5 Category 1B chemicals, 3 Category 1C chemicals, and 6 Category 1B/1C chemicals (Figure 2). Among the total chemical group, there were 76 liquids, 23 solids, and 1 gel (Figure 3). A total of 51 studies were reviewed to establish ChemSkin DB; 41 were used for in vivo data, consisting of 22 published papers, 14 reports, and 5 government documents. In vivo Draize scores and UN GHS/EU CLP classification information were also added. In addition, the in vitro data produced by the RhE test methods of OECD TG 439 were sourced from 16 published papers, 3 reports, and 4 government documents.

### 3.2. In Vivo Draize Scores of 86 Chemicals in ChemSkin DB

**Figure 4:**
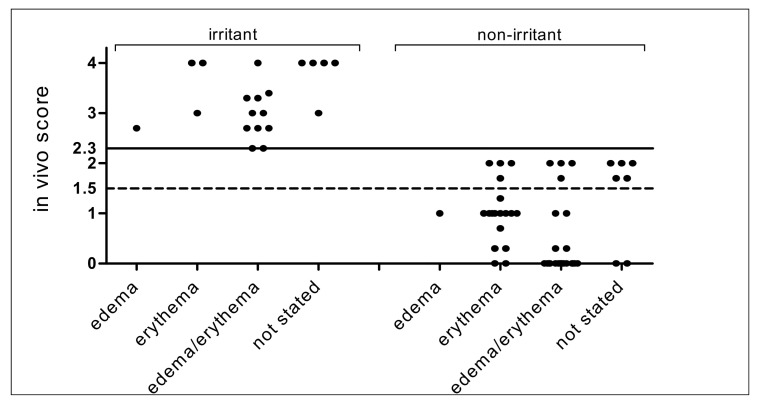
Scatter plot of edema/erythema in vivo Draize scores of 86 chemicals excluding Cat 1 chemicals according to irritant and non-irritant classification. The scatter plot shows the distribution of the in vivo Draize test scores. The value of 2.3 on the Y-axis indicates the in vivo score classification cut-off. The in vivo score 1.5 is marked to identify Cat 3.

**Figure 5:**
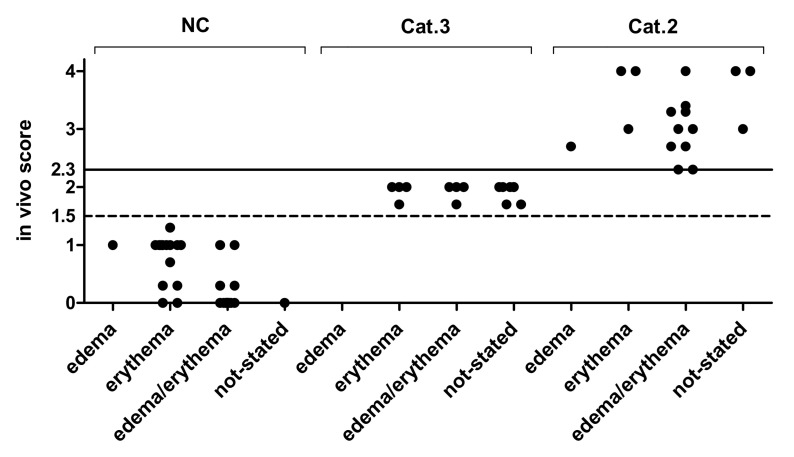
Scatter plot of edema/erythema in vivo scores of 86 chemicals excluding Cat 1 chemicals according to irritancy category (NC, Cat 3, and Cat 2).

Scatter plot is widely used to analyze the data distribution across categories [66]. In vivo Draize scores of 86 chemicals, excluding the 14 Cat 1 chemicals, are plotted as Figure 4 and Figure 5. Plotted chemicals based on irritant or non-irritant classification showed that some irritant chemicals have scores just above the threshold of 2.3, suggesting that they may be determined as false negatives (Figure 4). The mean ± SD of the in vivo score for each in vivo class was 3.27 ± 0.62 (20 irritants) for irritants (excluding Cat 1 chemicals, not stated values) and 0.78 ± 0.81 (55 non-irritants) for non-irritants (excluding not stated values) (irritant ≥ 2.3, non-irritant < 2.3), respectively. When Cat 3 is considered, the in vivo score of Cat 2 was 3.27 ± 0.62, Cat 3 was 1.92 ± 0.14 and No Cat was 0.36 ± 0.47 (Figure 5), which conforms to the current UN GHS classification criteria (UN GHS classification criteria: No Cat < 1.5, 1.5 ≤ Cat 3 < 2.3, 2.3 ≤ Cat 2 ≤ 4.0).

The scatter plot indicates the irritation signs for the Draize scoring. The in vivo score 2.3 is the cut-off for Cat 2 and 1.5 is the cut-off for Cat 3. Scores of NC chemicals without an in vivo score value available were set to 0.

### 3.3. Comparison of Prediction Results Produced by OECD TG 439 Test Methods for ChemSkin DB Substances

**Figure 6:**
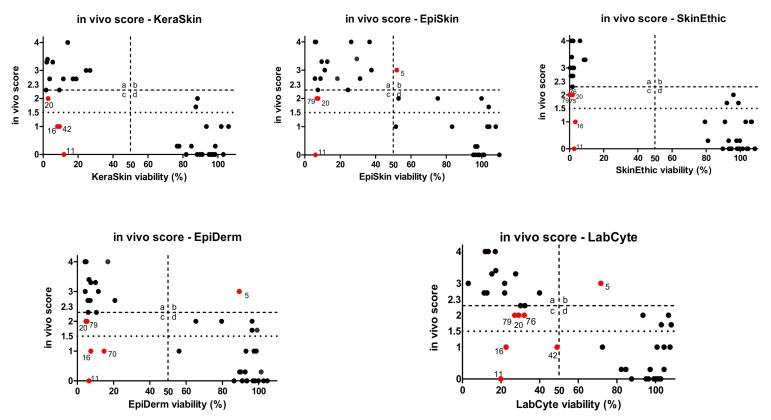
Relationship between in vivo scores and viability data of OECD TG 439 The cut-off of cell viability was defined as 50%, and the cut off in vivo score was defined as 2.3. The red dots are incorrectly predicted. In vitro irritant (I) = a, c area; in vitro non-irritant (NI) = b, d area; in vivo irritant (I) = a, b area; in vivo non-irritant (NI) = c, d area.

In the comparison of the results of the four approved methods of OECD TG 439 and in vivo scores, EpiSkin™ showed 3 false positives and 1 false negative, SkinEthic HCE™ showed 6 false positives, EpiDerm™ showed 5 false positives and 1 false negative, LabCyte EPI-MODEL24 showed 6 false positives and 1 false negative, and KeraSkin™ showed 4 false positives (Figure 6). Interestingly, compared with other models, KeraSkin™ and SkinEthic™ showed viability values near either 0% or 100%, reflecting that these models show a type of ‘all-or-none’ responses, while other models showed a number of borderline results around the cut-off.

We calculated the mean ± SD viability of irritants and non-irritants. KeraSkin™ showed 10.57 ± 9.12% and 77.55 ± 33.46% for irritants and non-irritants, respectively. EpiSkin™ showed 20.21 ± 14.16% and 88.23 ± 33.03%; SkinEthic HCE™ showed 2.74 ± 2.69% and 76.73 ± 40.42%; EpiDerm™ showed 13.48 ± 20.76% and 76.85 ± 34.44%; and LabCyte EPI-MODEL24 showed 22.44 ± 16.18% and 83.19 ± 29.72% for irritants and non-irritants, respectively.

The predictive capacity of VRMs is shown in Table 3. The sensitivity, specificity, and accuracy of EpiSkin™ were 97.1%, 79.3%, and 86.0%; those of SkinEthic™ were 97.1%, 69.4%, and 80.7%; those of LabCyte EPI-MODEL24 were 94.1%, 70%, and 77.2%; and those of KeraSkin™ were 100%, 82.2%, and 87.5%, respectively (Table 3). The sensitivity of OECD TG439 RhE SITs is in the order of KeraSkin™ 100% (20 out of 20 irritants were correctly identified), EpiSkin™ 97.1% (34 out of 35 irritants), SkinEthic™ 97.1% (33 out of 34 irritants), LabCyte EPI-MODEL24 94.1% (16 out of 17 irritants), and EpiDerm™ 89.5% (17 out of 19 irritants). The specificity is in the order of KeraSkin™ 82.2% (37 out of 45 non-irritants were correctly identified), EpiDerm™ 81.5% (44 out of 54 non-irritants), EpiSkin™ 79.3% (46 out of 58 non-irritants), LabCyte EPI-MODEL24 70% (28 out of 40 non-irritants), and SkinEthic™ 69.4% (34 out of 49 non-irritants). The RhE SITs mostly satisfied the OECD TG 439 PS criteria (sensitivity ≥ 80%, specificity ≥ 70%, and accuracy ≥ 75%) for the reference chemicals in ChemSkin DB outside of PS reference chemical lists.

The viability values for three Cat 3 chemicals in the OECD PS 20 reference substances, were 87.3%, 88.3%, and 2.9% for KeraSkin™; 104.0%, 99.9%, and 7.5% for EpiSkin™; 91.5–102.3%, 93.7–99.2%, and 1.2–1.7% for SkinEthic HCE™; 96.4%, 96.4%, and 4.7% for EpiDerm™; and 108.1%, 106.7%, and 29.1% for LabCyte EPI-MODEL24. These results confirm the inability of TG 439 to distinguish Cat 3 from No Cat or Cat 2.

A correlation analysis of the in vivo score and in vitro tissue viability was performed. The correlation coefficient was in the order of SkinEthic™ (−0.803), KeraSkin™ (−0.773), EpiSkin™ (absolute value of 0.760), LabCyte EPI-MODEL24 (−0.752), and EpiDerm™ (−0.749) (Table 4), suggesting that all VRMs and KeraSkin™ of OECD TG 439 produced tissue viability data highly correlated with in vivo scores.

## 4. Conclusions

In this study, we compiled the ChemSkin DB listing 100 reference chemicals for the development and evaluation of new test methods for skin irritation test through the review of more than 317 reference chemicals. The selection of correct reference chemicals is pivotal in the establishment and optimization of a new test method. Detailed information such as supporting literature, in vivo Draize scores, UN GHS/EU CLP classifications, and commercial sources were included, which could be invaluable for the developers of new skin irritation test methods. In addition, the test results produced by five methods approved in the current OECD Test No. 439 (2021) were included, compared in a table, a scatter plot, and analyzed for the correlation with in vivo Draize scores. Overall, the current RhE methods of TG 439 could not distinguish Category 3 from other categories, but strong correlations between viability and in vivo scores suggest an opportunity for further improvement. Collectively, we believe that our study will provide important insight into the future development of new in vitro skin irritation testing methods.

## Figures and Tables

**Table 1:** Primary Irritation Index (PII) and classification of skin irritancy.

Classification	Primary Irritation Index (PII)
Negligible	0–0.4
Slight Irritation	0.5–1.9
Moderate irritation	2–4.9
Severe irritation	5–8

**Table 2:** Information of major reference chemicals among substances included in ChemSkin DB.

c	Chemical	CAS No.	Physical State	In Vivo Category	In Vivo Score	Human In Vivo Result	In vivo Class	In Vivo Score Data Source References	KeraSkin^TM^ SIT	Vendor	VRMs				VRM References
											EpiSkin	SkinEthic	EpiDerm	LabCyte	
1	1-Decanol ^#, ##^	112-30-1	Liquid	Cat 2	2.3	NC	I	[29,30,31,32]	9.3	Sigma	7.3	1.4–2.4	6.0	32.3	[23,30,33,34]
2	Cyclamen aldehyde ^#^	103-95-7	Liquid	Cat 2	2.3	-	I	[30,31,32]	1.7	TCI	24.4	1.6–1.8	10.5	30.1	[23,30,33,34,35,36,37]
3	1-Bromohexane ^#, ###^	111-25-1	Liquid	Cat 2	2.7	I	I	[29,30,31,32]	18.9	Sigma	18.4	1.1–1.7	20.7	39.9	[23,30,33,34]
4	2-Chloromethyl-3,5-dimethyl-4-methoxypyridine HCl ^#^	86604-75-3	Solid	Cat 2	2.7	-	I	[30,31,32]	3.7	Sigma	5.7	0.7–0.8	6.7	12.7	[23,30,33,34]
5	Di-n-propyl disulphide ^#, ##^	629-19-6	Liquid	Cat 2	3.0	NC	I	[9,29,30,31,32]	26.8	Sigma	52.0	1.2–2.4	89.3	71.7	[23,30,33,34]
6	Potassium hydroxide (5% aq.) ^#^	1310-58-3	Liquid	Cat 2	3.0	-	I	[23,33,34]	−0.5	Sigma	37.7	0.1–0.6	4.3	3.0	[23,30,33,34]
7	Benzenethiol, 5-(1,1-dimethylethyl)-2-methyl ^#^	7340-90-1	Liquid	Cat 2	3.3	-	I	[23,33,34]	2.1	ACROS	12.7	8.5	10.0	27.6	[23,30,34,35]
8	1-Methyl-3-phenyl-1-piperazine ^#, ###^	5271-27-2	Solid	Cat 2	3.3	-	ci	[29,30,32]	5.5	AK Scientific	9.5	9.2	7.4	15.2	[23,30,34,35]
9	Heptanal ^#^	111-71-7	Liquid	Cat 2	3.4	I	I	[29,30,31,32]	2.5	Sigma	29.4	1.1–1.5	6.4	17.3	[23,30,34,35]
10	Tetrachloroethylene ^#^	127-18-4	Liquid	Cat 2	4.0	-	I	[30,31,32]	14.1	Sigma	26.2	0.6–1.5	4.8	17.0	[23,30,33,34]
11	1-Bromo-4-chlorobutane ^#^	6940-78-9	Liquid	NC	0	NC	NI	[29,30,31,32]	11.9	Sigma	6.0	1.6–4.9	6.4	19.8	[23,30,34,35]
12	Diethyl phthalate ^#^	84-66-2	Liquid	NC	0	-	NI	[30,31,32]	88.1	Sigma	95.3	86.8–93.1	86.4	87.6	[23,30,33,34]
13	Naphthalene acetic acid ^#^	86-87-3	Solid	NC	0	NC	NI	[30,31,32]	81.8	Sigma	96.4	95.5–99.9	104.8	99.7	[23,30,33,34]
14	Allyl phenoxyacetate ^#^	7493-74-5	Liquid	NC	0.3	-	NI	[30,31,32]	77.8	Sigma	96.5	67.9–92.0	92.6	82.3	[23,30,33,34]
15	Isopropanol ^#^	67-63-0	Liquid	NC	0.3	NC	NI	[29,30,31,32]	76.6	Sigma	97.5	91.3–104.1	90.1	84.6	[23,30,33,34]
16	4-Methylthio-benzaldehyde ^#^	3446-89-7	Liquid	NC	1.0	NC	NI	[9,29,31,32]	8.2	Sigma	51.5	2.8–4.2	7.4	22.6	[23,30,33,34]
17	Methyl stearate ^#^	112-61-8	Solid	NC	1.0	-	NI	[30,31,32]	93.4	Sigma	103.3	99.4–108.2	98.7	104.4	[23,30,33,34]
18	Heptyl butyrate ^#^	5870-93-9	Liquid	Cat 3	1.7	NC	NI	[29,30,31,32]	87.3	Sigma	104.0	91.5–102.3	96.4	108.1	[23,30,34,35,36]
19	Hexyl salicylate ^#^	6259-76-3	Liquid	Cat 3	2.0	NC	NI	[29,30,31,32]	88.3	Sigma	99.9	93.7–99.2	96.4	106.7	[23,30,34,35,36]
20	Cinnamaldehyde ^#^	104-55-2	Liquid	Cat 3	2.0	-	NI	[30,31,32]	2.9	Sigma	7.5	1.2–1.7	4.7	29.1	[23,30,33,34]
21	Nonanoic acid	112-05-0	Liquid	Cat 2	4.0	I	I	[9,29,37]	3.0	Sigma	4.6 *	1.1	–	–	[35,37]
22	Butyl methacrylate	97-88-1	Liquid	Cat 2	3.0	NC	I	[9,29,30,31,33]	24.6	Sigma	11.3 (5.7) *	1.1–3.7	11.6	24.5–33.6	[30,33,34,35,36]
23	Butyric acid	107-92-6	Liquid	Cat 1B	-	-	I	[33,38]	2.2	Sigma	2.1–4.5	0.4–1.1	–	–	[33,38]
24	Decanoic acid (capric acid)	334-48-5	Liquid	Cat 2	4.0	I	I	[9,29,30]	43.6	Sigma	3.1	<10	5.5	6.1–17.6	[30,34,35,39]
25	1-Bromopentane	110-53-2	Liquid	Cat 2	2.7	-	I	[30,31]	11.6	Sigma	31.2 (9.9–45.1) *	<10 (2.0) ^※^	5.8	17.7–24.3	[30,31,34,37,38,39,40]
26	alpha-Terpineol	98-55-5	Liquid	Cat 2	2.7	NC	I	[9,29,30,31]	17.1	Sigma	8.9 (2.8–15.2) **	<10 (1.3–3.0) ^※※^	7.1 (17.9–70.9) **	7.3–14.5	[30,31,34,36,39]
27	Heptanoic acid	111-14-8	Liquid	Cat 1B	(PII 5.6)	I	I	[29,39,41]	2.2	Sigma	–	<10	–	–	[39]
28	Octanoic acid (caprylic acid)	124-07-2	Liquid	Cat 1B/1C	(PII 4.4)	I	I	[29,31,38,42,43]	2.0	Sigma	4.5–6.1	0.5–1.1	–	–	[33,38,39]
29	*N,N-*Dimethylisopropylamine	996-35-0	Liquid	Cat 1B/1C	(PII 5.6)	-	I	[33,38,42]	–	Sigma	6.3–8.4	0.4–1.3	–	–	[33,38]
30	Polyethylene glycol 400 (PEG-400)	25322-68-3	Liquid	NC	0	NC	NI	[29,30,34,39]	96.1	TCI	101.4 ***	>80 (93.4) ***	99.9	98.2–106.6 (98.2) ***	[30,34,39,44]
31	3-(Chloropropyl) trimethoxysilane (Silane A-1430)	2530-87-2	Liquid	NC	0	-	NI	[31,35,45]	3.3	Sigma	36.0–94.6 (10.8) *	80.7	61.7–98.6	–	[33,35,37,38]
32	3,3′-Dithiodipropionic acid	1119-62-6	Solid	NC	0	-	NI	[30,31,45]	96.3	Sigma	96.7–107.5	102.4–117.1	98.0	89.9–100	[30,33,34,38]
33	2-Phenylethanol (phenylethyl alcohol)	60-12-8	Liquid	NC	1.0	-	NI	[31,35]	0.3	Sigma	63.1–104.4 (91.7) *	5.6	45.7–92.9	–	[30,35,37,38]
34	Benzyl salicylate	118-58-1	Liquid	NC	0.3	NC	NI	[29,30,45]	98.5	Sigma	121.37 (96.4) *	86.5–113.3	89.5	93.6–99.9	[31,33,34,37]
35	1-(4-Chlorophenyl)-3-(3,4-dichlorophenyl) urea	101-20-2	Solid	NC	0	-	NI	[46,47]	102.3	Sigma	111.2	–	–	–	[46]
36	3,3-Dimethylpentane	562-49-2	Liquid	NC	0	-	NI	[30,34]	88.3	ACROS	–	–	102.4	72.3–90.8	[30,34]
37	4,4′-Methylenebis(2,6-di-tert-butylphenol)	118-82-1	Solid	NC^u^	0	-	NI	[30,31]	97.3	Sigma	85.8–111.2	86.5–113.3	96.9 (94.4) **	100.0–101.3	[30,31,33,34,38]
38	Dodecanoic acid (lauric acid)	143-07-7	Solid	NC	0.3	NC	NI	[29,30,31]	84.8	Sigma	102.5–127.6	78.0–102.1	20.2 (101.4) **	94.0–110.0	[30,31,33,34]
39	1-Chloro-3-nitrobenzene (3-chloronitrobenzene)	121-73-3	Solid	NC	0	-	NI	[30,31]	87.7	Sigma	90.0 (102.5) *	–	96.9	95.2–104.3	[30,31,34,38]
40	Benzyl benzoate	120-51-4	Liquid	NC	0	-	NI	[30,31]	98.3	Sigma	110.0	84.3–104.1	93.4 (100.1) **	99.6–105.7	[30,31,33,34]
41	2-(Formylamino)-3-thiophenecarboxylic acid	43028-69-9	Solid	NC	0	-	NI	[31,45]	100.9	Sigma	89.0–95.8	108.0	97.8–105.7	–	[31,35]
42	Sodium bisulphite	7631-90-5	Solid	NC	1.0	-	NI	[30,34,45]	9.4	Sigma	108.0	79.1–99.7	56.1	11.1–74.7	[30,33,34]
43	4-Acetoxy-2,5-dimethyl-3(2H)-furanone (2,5-dimethyl-4-oxo-4,5-dihydrofuran-3-yl acetate)	4166-20-5	Liquid	NC	0	-	NI	[31,35,45]	4.6	TCI	98.2–114.3	102.3	80.3–91.2	–	[31,35]
44	4-Amino-4H-1,2,4-triazole (4-amino-1,2,4-triazole)	584-13-4	Solid	NC	0	-	NI	[30,31,45]	89.7	Sigma	98.3	102.2–106.0	91.0 (92.1) **	97.4–101.0	[30,31,34]
45	Dipropylene glycol	25265-71-8	Liquid	NC	0	NC	NI	[9,30,31,45]	94.9	Sigma	81.5–111.5	95.9–103.5	93.5	92.9–109.9	[30,33,34,38]
46	Dipropylene glycol butyl ether, mixture of isomers (dipropylene glycol monobutyl ether; DPnB)	29911-28-2	Liquid	NC	0	-	NI	[31,45]	13.9	Sigma	85.0–113.9	97.4	101.2	–	[33,35,38]
47	Erucamide	112-84-5	Solid	NC	0	-	NI	[30,31,45]	103.1	Sigma	97.8–106.2	95.1–107.7	102.7	90.0–102.5	[30,33,35,38]
48	Propylene glycol	1-6	Liquid	NC	0	NC	NI	[29,39,48,49]	84.5	Sigma	–	>80	–	–	[39]
49	Triethylene glycol	112-27-6	Liquid	NC	0	-	NI	[31,35,45]	76.6	Sigma	90.7–116.9 (101.9) *	101.4	95.0–95.3	–	[31,33,35,37,38]
50	Sodium bicarbonate	144-55-8	Solid	NC	0	-	NI	[30,31,45]	91.0	Sigma	92.0–104.2	94.7–113.3	90.9	99.6–100.3	[30,33,38]
51	Isopropyl palmitate	142-91-6	Liquid	NC	1.0	NC	NI	[29,30,31,45]	106.0	Sigma	93.9–104.1	100.7–115.1	93.0	102.5–115.9	[30,33,34,38]
52	Isopropyl myristate	110-27-0	Liquid	NC	1.0	NC	NI	[29,30,31,45]	101.9	Sigma	102.9–118.6	98.6–110.5	97.5	97.2–107.9	[30,33,34,38]
53	2-Ethylhexyl 4-methoxycinnamate (octinoxate)	5466-77-3	Liquid	NC	-	-	NI	[50,51]	106.5	Sigma	–	83.3–109.7	–	–	[50]
54	Tetrabromophenol blue	4430-25-5	Solid	NC	-	-	NI	[46,52,53]	89.9	Sigma	109.8 (110.8) ****	–	–	–	[46,52]
55	Piroctone olamine	68890-66-4	Solid	Cat 2	-	-	I	[52,54]	1.7	TCI	6.0 (7–11) *****	–	–	–	[52,54]
56	25% Cetyltrimethylammonium chloride solution (in D.W.) (cetrimonium chloride)	112-02-7	Liquid	Cat 2	-	-	I	[52,54,55]	1.1	Sigma	10.5	–	–	–	[52]
57	2-Hydroxy-4-methoxybenzophenone, (Benzophenone-3)	131-57-7	Solid	NC	-	-	NI	[52,54,56]	99.3	Sigma	104.1 (64–119) *****	–	–	–	[52,54]
58	Cyclohexadecanone	2550-52-9	Solid	NC	0	-	NI	[31,45]	103.9	Symrise	112.4–121.9	–	7.9–113.6	–	[31]
59	Methyl laurate	111-82-0	Liquid	Cat 3	2.0	NC	NI	[29,31,45]	91.2	Sigma	83.9 *	>80	103.3	–	[31,37,39]
60	Capryl-isostearate	209802-43-7	Liquid	NC	1.0	-	NI	[31,45]	94.1	Nikko Chemical	96.0–102.3	–	99.5–108.0	–	[31]
61	2-Ethylhexyl-4-aminobenzoate	26218-04-2	Solid	NC	0.7	-	NI	[31,45]	103.1	Santacruz	90.9–111.4	–	91.9–107.3	–	[31]
62	2-Phenylhexanenitrile	3508-98-3	Liquid	Cat 3	1.7	-	NI	[31,45]	76.0	IFF	86.7–116.2	–	73.9–82.1	–	[31]
63	Barium sulfate	7727-43-7	Solid	NC	-	-	NI		94.8	ACROS	95.3	99.4	99.0	92.9	[44]
64	Diisopropyl sebacate	7491-02-3	Liquid	NC	-	-	NI		93.8	Nippon Fine Chemicals	91.1	91.0	–	104.6	[44]
65	10% Xanthan gum (in D.W.)	11138-66-2	Gel	NC	-	-	NI	[44,57,58]	99.6	Sigma	101.3	98.4	–	94.0	[44]
66	3-Chloro-4-fluoronitrobenzene	350-30-1	Solid	NC	1.0	-	NI	[31,45]	80.7	Sigma	11.0–54.9 (5.0) *	101.6	53.2–101.7	–	[31,35,37]
67	1,5-Hexadiene	592-42-7	Liquid	NC	0	-	NI	[30,34]	–	Sigma	–	–	100.4	85.6–95.6	[30,34]
68	Glycerol	56-81-5	Liquid	NC	0	-	NI	[30,34]	–	Sigma	–	–	99.7	98.1–125.7	[30,34]
69	Benzyl acetate	140-11-4	Liquid	NC	1.0	-	NI	[30,31,45]	–	Sigma	21.7	–	74.4 (102.8) **	11.3–37.4	[30,31,34]
70	Hydroxycitronellal	107-75-5	Liquid	NC	1.0	NC	NI	[9,30,45]	–	Sigma	83.3	79.3	14.7	19.8–32.3	[30,33,34,35,38]
71	n-Butyl propionate	590-01-2	Liquid	NC	1.0	-	NI	[30,31,45]	–	Sigma	92.1–104.0 (31.5) ^※※※^	–	20.8 ^※※※^ (108.7) **	23.2–45.9	[30,31,34,38]
72	Benzyl alcohol	100-51-6	Liquid	NC	1.3	NC	NI	[29,30,31]	–	Sigma	72.5 ^※※※^	<10	5.5 ^※※※^ (89.2) **	5.6–17.8	[30,31,34,39]
73	Allyl heptanoate	142-19-8	Liquid	Cat 3	1.7	-	NI	[31,33,38]	–	Sigma	88.6–112.5 (101.1) ^※※※^	74.4–101.7	97.0–102.1 (99.2) ^※※※^	95.6–108.9	[30,31,33,34,38]
74	2-Ethoxy ethyl methacrylate	2370-63-0	Liquid	Cat 3	1.7	-	NI	[31,38,45]	–	Sigma	65.3–116.3 (6.7) ^※※※※^	–	6.9 ^※※※^ (99.0) **	29.3–74.4	[30,31,33,34,38,46]
75	Linalyl acetate	115-95-7	Liquid	Cat 3	2.0	NC	NI	[29,30,31]	–	Sigma	50.0–103.8 (43.2) ^※※※^	1.25	79.6	86.8–101.2	[30,34,35,38]
76	Terpinyl acetate	80-26-2	Liquid	Cat 3	2.0	NC	NI	[29,30,31]	–	Sigma	4.9–75.4 (53.0) ^※※※^	1.7–3.2	65.4	26.2–36.3	[30,33,34,38]
77	Linalool	78-70-6	Liquid	Cat 3	2.0	-	NI	[30,31,45]	–	Sigma	14.7	–	4.7	7.9–25.4	[30,34]
78	D-Limonene	5989-27-5	Liquid	Cat 3	2.0	-	NI	[31,45]	–	Sigma	–	1.1	10.4–23.6 (16.3) **	–	[31,40,59]
79	Eugenol	97-53-0	Liquid	Cat 3	2.0	NC	NI	[29,30,31,38]	–	Sigma	5.1–8.3	0.0–0.1	5.26	18.5–32.2	[30,33,34,38]
80	Methyl palmitate ^####^	112-39-0	Liquid	Cat 2	3.0	NC	I	[29,31,33,45]	–	Sigma	100.5–104.9	70.9–110.6	95.7	–	[31,33,38]
81	1,1,1-Trichloroethane	71-55-6	Liquid	Cat 2	4.0	-	I	[30,31,45]	–	Sigma	36.6	<20 (6.1) ^※^	16.8	10.1–13.4	[30,34,39,40]
82	SLS (50% aq.)	151-21-3	Liquid	Cat 2	4.0	-	I	[31,33]	–	Sigma	13.1–34.5 (2.3) *	0.7–1.1 (1.6) ^※^	11.9	–	[31,33,37,38,40]
83	SLS (20% aq.)	151-21-3	Liquid	Cat 2	4.0	I	I	[29,30,31,33]	–	Sigma	5.2–8.3 (61.3) ^※※※^	1.0–1.7	4.2 (13.2) **	9.4–13.4	[23,30,31,33,34,38]
84	SLS (5% aq.)	151-21-3	Liquid	Cat 2	4.0	-	I	[30,34]	–	Sigma	5.8 ***	2.1 ***	4.3	11.4–14.5	[30,34,44]
85	Tri-isobutyl phosphate	126-71-6	Liquid	Cat 3	2.0	-	NI	[24,30,36,38,45]	–	Santa Cruz Biotechnology	4.4–8.3 (5.9–7.1) **	1.3–1.7	6.4–10.6 (24.3–44.6) **	–	[30,31,36,38]
86	10-Undecenoic acid	112-38-9	Solid (Liquid at room temp.)	Cat 3	2.0	NC	NI	[29,31,45,60]	–	Sigma	6.0–15.3 (6.2) *	2.7–14.1	10.0–17.5 (13.2) **	–	[31,38,40,59,60]
87	*dl*-Citronellol	106-22-9	Liquid	Cat 3	2.0	NC	NI	[29,31,45]	–	Sigma	5.9–11.4	0.3–1.0	9.7–12.3 (11.1) **	–	[31,40,59,61]
88	33% Sodium undecylenate (in aqueous solution)	3398-33-2	Liquid	Cat 3	(PII 1.7)	-	NI	[33,42]	–	Sigma	9.4–28.0	0.8–1.7	–	–	[33,38]
89	2-Methoxyphenol (guaiacol)	90-05-1	Liquid	Cat 3	(PII 2.4)	-	NI	[33,40,60]	–	Sigma	0.7	0.5–0.6	–	–	[33,60]
90	1,9-Decadiene	1647-16-1	Liquid	Cat 3	(PII 3.0)	-	NI	[33,42,60]	-	Sigma	17.2–20.0 (10.6) *	1.3–2.3	–	–	[33,38,60]
91	2-*tert*-Butylphenol	88-18-6	Liquid	Cat 1B/1C	(PII 5.6)	-	I	[38,42,62,63]	-	Sigma	3.1–5.3	1.4–9.5	–	–	[33,38]
92	Carvacrol	499-75-2	Liquid	Cat 1B/1C	(PII > 4)	-	I	[38,42,62]	-	Sigma	4.5–5.6	0–27.1	–	–	[33,38]
93	Cyclohexylamine	108-91-8	Liquid	Cat 1B/1C	-	I	I	[33,38,64]	-	Sigma	4.3–6.6 (3.6) *	0.5–1.9	–	–	[33,38,60]
94	Lactic acid	50-21-5 (598-82-3)	Liquid	Cat 1B/1C	-	I	I	[29,33,60,63]	-	Sigma	8.4	0.5–1.5	–	–	[33,60]
95	Ethanolamine	141-43-5	Liquid	Cat 1B	-	-	I	[33,60]	-	Sigma	3.4	0.2–21.2	–	–	[33,60]
96	Boron trifluoride acetic acid complex	373-61-5	Liquid	Cat 1B	-	-	I	[33,38]	-	Sigma	3.4–4.5	0.4–1.0	–	–	[33,38]
97	Propionic acid	79-09-4	Liquid	Cat 1B	-	-	I	[33,38,60]	-	Sigma	3.0–5.5 (2.9) *	0.6–0.7	–	–	[33,38,60]
98	*N,N-*Dimethylbenzylamine	103-83-3	Liquid	Cat 1C	>4	-	I	[33,38,60,65]	-	Sigma	4.7–6.8 (0.3) *	0.5–0.9	–	–	[33,38,60]
99	Maleic anhydride	108-31-6	Solid	Cat 1C	-	I	I	[38,60,64]	-	Sigma	4.7–7.5 (6.1) *	0.4–0.5	–	–	[33,38,60]
100	48% Fluoroboric acid (in D.W.) (hydrogen tetrafluoroborate)	16872-11-0	Liquid	Cat 1C	-	-	I	[33,60]	-	Sigma	2.4	0.6–1.1			[33,60]

VRMs: Validated reference methods; KeraSkin: KeraSkin^TM^ SIT; EpiSkin: EpiSkin^TM^ SIT; SkinEthic: SkinEthic^TM^ RHE; EpiDerm: EpiDerm^TM^ SIT; LabCyte: LabCyte EPI-MODEL 24 SIT; PII: primary irritation index; SLS: sodium dodecyl sulfate. Abbreviations: VRMs = in vitro cell viability value, % of control; NC = No category, skin non-irritant; I = skin irritant; Cat 2 = UN GHS Category 2, skin irritant; Cat 1B = UN GHS Sub-category 1B, skin corrosive; Cat 1B/1C = combination of UN GHS sub-category 1B and 1C, skin corrosive. Cat 3 = UN GHS optional Category 3, skin mild irritant (in vivo score 1.5 ≤ Cat 3 < 2.3). OECD TG 439 (in vitro skin irritation: RhE test methods) does not classify chemicals to the optional Category 3 [19,32]. Under this test guideline, Cat 3 is considered as no category (non-irritant). ^#^ Twenty reference chemicals are performance standards (PS) based on the *Series on Testing and Assessment No. 220* [32]. These PS are now available related to the present OECD TG 439. PS are available to facilitate the validation and assessment of similar and modified RhE-based test methods, in accordance with the principles of OECD Guidance Document No. 34 [7]. ^##^ 1-Decanol (a borderline reference chemical) and di-n-propyl disulphide (a false negative of the VRM) are non-irritants in humans, although being identified as irritants in the rabbit test. Since RhE models are based on cells of human origin; they may predict these reference chemicals as non-irritants (UN GHS No category). ^###^ According to the OECD TG 439, 1-methyl-3-phenyl-1-piperazine and 1-bromohexane can have variable results in different laboratories dependent on the supplier. ^####^ According to the *Series on Testing and Assessment No. 137* [31], methyl palmitate is a false negative in EpiSkin^TM^, modified EpiDerm^TM^, and SkinEthic^TM^ RHE. This chemical is also a non-irritant to humans based on the human 4-hr patch test. * EpiSkin^TM^ data produced in China [37]; ** Data based on the *Series on Testing and Assessment No. 137* [31]; *** Data reported by Sugiyama et al., 2018 [44]; **** Data reported by Alépée et al., 2016 [52]; ***** SCCS Memorandum (addendum) data [54] on the in vitro EpiSkin^TM^. ^※^ Data reported by Kandárová et al., 2006 [40]; ^※※^ Data reported by Alépée et al., 2010 [36]; ^※※※^ Data reported by Kandárová et al., 2009 [30]; ^※※※※^ Data reported by Alépée et al., 2015b [33].

**Table 3:** Predictive capacity of VRM viability.

	KeraSkin^TM^		EpiSkin^TM^		SkinEthic^TM^		EpiDerm^TM^		LabCyte EPI-MODEL24		PS
	I	NI	I	NI	I	NI	I	NI	I	NI	
I	20	0	34	1	33	1	17	2	16	1	
NI	8	37	12	46	15	34	10	44	12	28	
Total (n)		65		93		83		73		57	
Sensitivity		100%		97.1%		97.1%		89.5%		94.1%	80%
Specificity		82.2%		79.3%		69.4%		81.5%		70.0%	70%
Accuracy		87.5%		86.0%		80.7%		83.6%		77.2%	75%

**Table 4:** Correlation between in vivo score and VRM viability.

	In Vivo Score	KeraSkin^TM^	EpiSkin^TM^	SkinEthic^TM^ RHE	EpiDerm^TM^	LabCyte EPI-MODEL24
In vivo score	1.000					
KeraSkin^TM^	−0.773 *	1.000				
EpiSkin^TM^	−0.760 *	0.899 *	1.000			
SkinEthic^TM^ RHE	−0.803 *	0.938 *	0.957 *	1.000		
EpiDerm^TM^	−0.749 *	0.940 *	0.933 *	0.933 *	1.000	
LabCyte EPI-MODEL24	−0.752 *	0.960 *	0.919 *	0.938 *	0.978 *	1.000

* Correlation is significant at the 0.01 level (2-tailed).

## Data Availability

Not applicable.
